# Cyclo-C stabilizes PEX13 to inhibit porcine epidemic diarrhea virus replication by blocking pexophagy-mediated disruption of antiviral innate immunity

**DOI:** 10.1128/jvi.00785-26

**Published:** 2026-07-17

**Authors:** Jinxiu Lou, Zhiwei Guo, Kang Chen, Yuanmingyue Tian, Zhenpeng Tang, Zhendong Xin, Ping Jiang, Gongguan Liu, Xianwei Wang

**Affiliations:** 1 Key Laboratory of Animal Disease Diagnostics and Immunology, Ministry of Agriculture, MOE International Joint Collaborative Research Laboratory for Animal Health & Food Safety, College of Veterinary Medicine, Nanjing Agricultural University 261674, Nanjing, China; 2 Jiangsu Co-Innovation Center for Prevention and Control of Important Animal Infectious Diseases and Zoonoses, Yangzhou, China; University of Kentucky College of Medicine, Lexington, Kentucky, USA

**Keywords:** neonatal piglets, host-directed antiviral drugs, Cyclo-C, PEX13, pexophagy, type III interferon, porcine coronavirus

## Abstract

The persistent threat of porcine epidemic diarrhea virus (PEDV) to the global swine industry is compounded by high neonatal piglet mortality and the absence of effective antiviral therapies. Host-directed strategies that reinforce immunity offer a promising avenue to counter viral immune evasion. Through screening of an FDA-approved compound library, we identify the small-molecule cyclocytidine hydrochloride (Cyclo-C) as a potent inhibitor of PEDV replication that acts by stabilizing the peroxisomal biogenesis factor PEX13, a previously unrecognized host restriction factor. The antiviral activity of Cyclo-C is strictly PEX13-dependent, as it is completely abrogated in PEX13 knockout cells. Mechanistically, Cyclo-C disrupts the interaction between PEX13 and the viral nonstructural protein 8 (NSP8), thereby preventing NSP8-mediated PEX13 degradation and the subsequent induction of PI3K/AKT/mTOR-driven pexophagy. Preservation of peroxisomal integrity stabilizes the peroxisome-localized pool of MAVS, leading to a robust enhancement of type III interferon (IFN-III) responses that suppress viral replication. Critically, this mechanism translates *in vivo*, where Cyclo-C treatment of PEDV-challenged piglets significantly reduces mortality, lowers viral loads, and protects intestinal villus architecture. Our findings establish Cyclo-C as a first-in-class host-directed therapeutic candidate and validate the concept that pharmacological preservation of peroxisome-mediated innate immunity represents an effective antiviral strategy against enteric coronaviruses.

**IMPORTANCE:**

The high genetic variability of porcine epidemic diarrhea virus (PEDV) limits current vaccine efficacy, and no antiviral therapeutics exist. Host-directed therapies targeting cellular pathways that viruses exploit for immune evasion offer an alternative approach. Here, we identify the FDA-approved compound Cyclo-C as a potent inhibitor of PEDV replication. Cyclo-C acts by stabilizing PEX13, a host protein that the virus degrades to evade immunity. By blocking viral protein NSP8 from binding PEX13, Cyclo-C prevents virus-induced pexophagy, thereby preserving peroxisomal integrity. This preserves peroxisome-localized MAVS and enhances type III interferon responses. In infected neonatal piglets, Cyclo-C reduced mortality and viral loads while protecting intestinal integrity. This study provides proof of concept that targeting peroxisomal immune regulation is a viable antiviral strategy and identifies Cyclo-C as a promising candidate for treating PEDV infection.

## INTRODUCTION

Coronaviruses constitute a large family of enveloped RNA viruses that pose significant threats to both human and animal health, with their high mutability and transmissibility rendering them a persistent global concern (1). In the swine industry, coronaviruses rank among the most devastating pathogens, causing substantial economic losses and threatening food security worldwide (2). The predominant coronaviruses circulating on pig farms include porcine epidemic diarrhea virus (PEDV), transmissible gastroenteritis virus (TGEV), and porcine deltacoronavirus (PDCoV). PEDV, a member of the genus *Alphacoronavirus*, poses a severe threat to the global swine industry, causing high mortality rates in neonatal piglets and substantial economic losses (3). The virus primarily infects porcine intestinal epithelial cells, leading to acute vomiting, watery diarrhea, and dehydration (4, 5). Due to the emergence of the epidemic G2c variant and the high variability of its spike (S) protein (6–9), and considering that the immature immune system of neonatal piglets limits the efficacy of direct vaccination, currently available commercial vaccines provide suboptimal protection for pig herds (10). This underscores the urgent need to develop more effective antiviral therapeutics.

The innate immune system, particularly the type III interferon (IFN-III or IFN-λ) response, constitutes the first line of defense against viral pathogens at mucosal surfaces such as the intestinal epithelium (11–14). Mitochondrial antiviral-signaling protein (MAVS) serves as a central adaptor in the RIG-I-like receptor (RLR) pathway and is essential for initiating interferon production following viral RNA recognition (15). Notably, in intestinal epithelial cells, a distinct pool of MAVS localized to peroxisomes is critical for the rapid induction of IFN-III, which plays a pivotal role in controlling enteric viral infections (16). For example, viruses such as hepatitis C virus (HCV) and human cytomegalovirus (HCMV) employ viral proteins to directly target and cleave peroxisomal MAVS, resulting in its degradation and functional impairment (17, 18). Furthermore, infection with flaviviruses such as dengue virus (DENV) and West Nile virus (WNV) reduces peroxisome abundance and consequently inhibits type III interferon expression (19). These findings highlight that peroxisomes are not merely metabolic organelles but also function as essential signaling platforms for antiviral innate immunity (20).

To establish productive infection, PEDV, like many other viruses, has evolved sophisticated strategies to evade and suppress host IFN responses (21–25). Our previous work identified a novel immune evasion mechanism employed by PEDV, whereby the viral nonstructural protein 8 (NSP8) directly interacts with PEX13, a core component of the peroxisomal protein import machinery (26). This interaction promotes PEX13 downregulation, triggering pexophagy, the selective autophagic degradation of peroxisomes (26). Consequently, the peroxisomal pool of MAVS is depleted, leading to suppression of IFN-III-mediated antiviral signaling and the establishment of a cellular environment favorable for viral replication (26, 27). These findings identify PEX13 as a critical host factor at the intersection of peroxisome biology, innate immunity, and viral pathogenesis. We therefore hypothesized that targeting and stabilizing PEX13 represents a promising host-directed therapeutic strategy to counteract PEDV infection by restoring peroxisome-dependent antiviral signaling and overcoming viral immune evasion.

Traditional antiviral strategies primarily target viral components, such as polymerases or proteases (28, 29). However, the rapid mutation rates of RNA viruses frequently lead to the emergence of drug resistance (30, 31). Host-targeted antiviral drugs focus on cellular factors essential for viral replication rather than viral proteins themselves, which offers several compelling advantages, including a higher genetic barrier to resistance and the potential for broad-spectrum antiviral activity (32). PEDV replication depends extensively on host cellular machinery, making it an ideal candidate for host-targeted intervention strategies. Recent research has identified multiple host dependency factors critical for PEDV infection, including cathepsins for viral entry (33, 34), the unfolded protein response (UPR) pathway for replication complex formation (35), and autophagy machinery for cytoplasmic double-membrane vesicle generation (36, 37). However, no drug screening campaigns targeting the peroxisomal pathway have been reported.

Here, we identify the FDA-approved compound cyclocytidine hydrochloride (Cyclo-C) as a first-in-class host-directed inhibitor of PEDV replication that acts by stabilizing the peroxisomal biogenesis factor PEX13. Mechanistically, Cyclo-C disrupts NSP8-PEX13 interaction, preventing NSP8-mediated PEX13 degradation and subsequent pexophagy, thereby preserving peroxisomal MAVS and potentiating type III interferon responses to suppress viral replication. Critically, Cyclo-C confers protection against PEDV challenge in piglets and exhibits inhibitory activity against TGEV and PDCoV. These findings establish pharmacological targeting of PEX13 as an effective antiviral strategy and identify Cyclo-C as a promising host-directed therapeutic candidate for combating enteric coronavirus infections.

## RESULTS

### PEX13 exerts broad-spectrum antiviral activity against swine enteric coronaviruses

**Fig 1:**
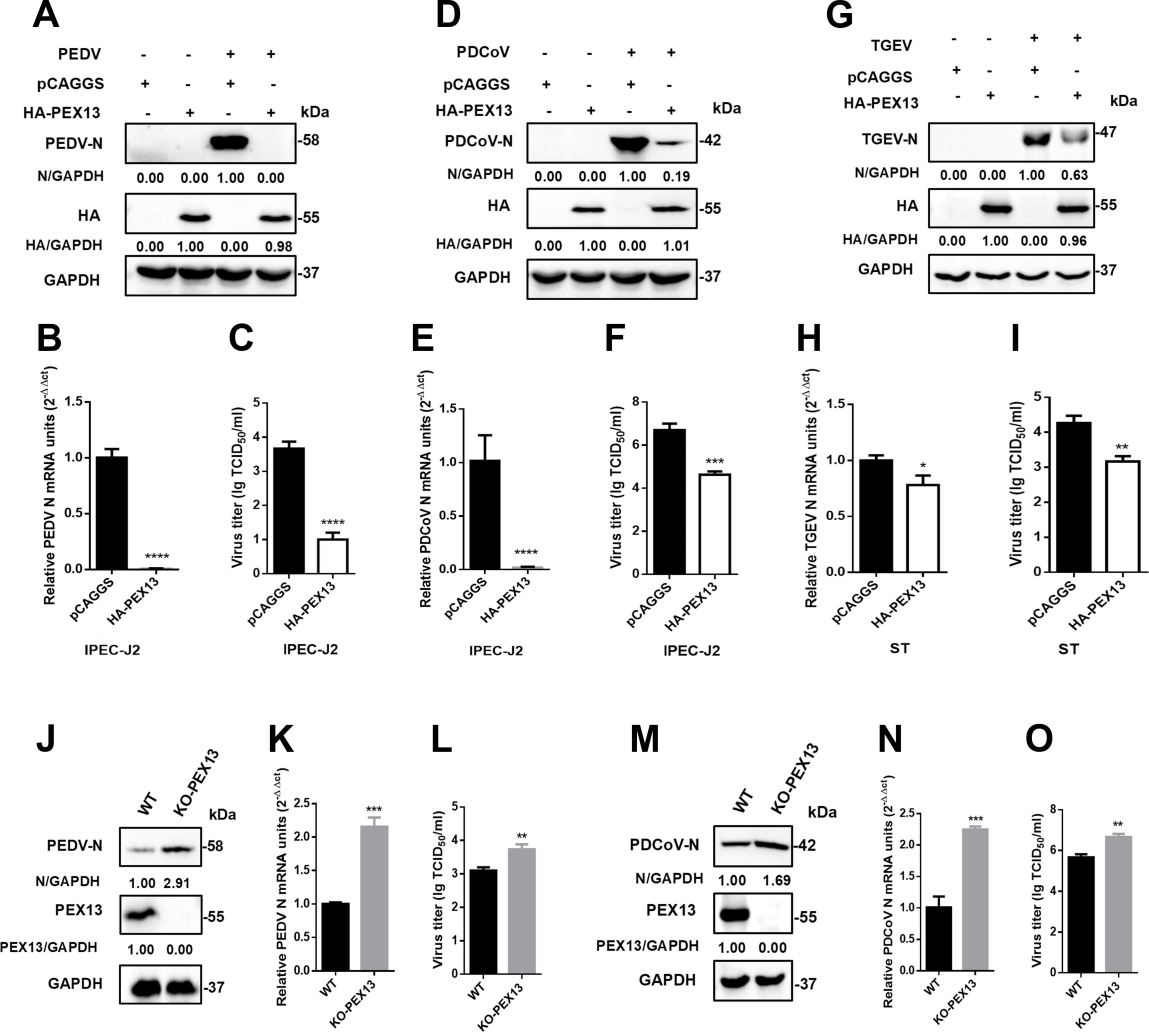
PEX13 exhibits antiviral activity against diverse swine enteric coronaviruses. (**A–C**) Overexpression of PEX13 inhibits PEDV replication in IPEC-J2 cells. Cells were transfected with pCAGGS-HA-PEX13 or an empty vector for 24 h and then infected with PEDV (MOI = 1). At 24 h post-infection (hpi), cells were harvested for Western blotting (**A**), RT-qPCR (**B**), and TCID_50_ assays (**C**). Data are means ± SD from three independent experiments. ****, *P* < 0.0001. (**D–F**) Overexpression of PEX13 suppresses PDCoV replication in IPEC-J2 cells. Cells were transfected as described above and infected with PDCoV (MOI = 0.5). Samples were collected at 24 hpi for Western blotting (**D**), RT-qPCR (**E**), and TCID_50_ assays (**F**). Data are means ± SD from three independent experiments. ***, *P* < 0.001; ****, *P* < 0.0001. (**G–I**) Overexpression of PEX13 inhibits TGEV replication in ST cells. ST cells were transfected with pCAGGS-HA-PEX13 or an empty vector for 24 h and infected with TGEV (MOI = 0.5). Samples were collected at 24 hpi for Western blotting (**G**), RT-qPCR (**H**), and TCID_50_ assays (**I**). Data are means ± SD from three independent experiments. *, *P* < 0.05; **, *P* < 0.01. (**J–L**) PEX13-deficient IPEC-J2 cells exhibit increased susceptibility to PEDV infection. Wild-type (WT) and PEX13 knockout (PEX13-KO) IPEC-J2 cells were infected with PEDV (MOI = 1). Samples were collected at 24 hpi for Western blotting (**J**), RT-qPCR (**K**), and TCID_50_ assays (**L**). Data are means ± SD from three independent experiments. **, *P* < 0.01; ***, *P* < 0.001. (**M–O**) PDCoV infection is enhanced in PEX13-KO IPEC-J2 cells. WT and PEX13-KO IPEC-J2 cells were infected with PDCoV (MOI = 1). Cells were collected at 24 hpi for Western blotting (**M**), RT-qPCR (**N**), and TCID_50_ assays (**O**). Data are means ± SD from three independent experiments. **, *P* < 0.01; ***, *P* < 0.001.

Our previous studies demonstrated that PEDV targets the PEX13 protein to induce pexophagy, thereby suppressing IFN-III production and facilitating viral replication (26). Based on these findings, we hypothesized that PEX13 may serve as a promising host-targeted antiviral factor against PEDV infection. To explore this, IPEC-J2 cells were transfected with an HA-PEX13 expression plasmid and subsequently infected with PEDV for 24 h. Cells were then harvested for Western blotting, RT-qPCR, and viral titration. Notably, overexpression of PEX13 almost completely abrogated PEDV N protein expression (Fig. 1A) and resulted in a marked reduction in both viral RNA levels and progeny virus titers (Fig. 1B and C).

To assess whether the antiviral effect of PEX13 extends to other swine enteric coronaviruses, we evaluated its effects on PDCoV and TGEV. HA-PEX13–transfected cells were infected with PDCoV or TGEV, followed by analysis of viral protein expression, genomic RNA levels, and viral titers. Overexpression of PEX13 significantly suppressed replication of both PDCoV and TGEV (Fig. 1D through I), indicating a broad-spectrum antiviral activity.

To further corroborate these findings, we generated PEX13 knockout (PEX13-KO) IPEC-J2 cells using CRISPR-Cas9 technology. WT and PEX13-KO cells were infected with PEDV and harvested at 24 hpi. Loss of PEX13 significantly enhanced PEDV N protein expression, viral RNA levels, and viral titers compared to WT controls (Fig. 1J through L). Similarly, PEX13-KO cells infected with PDCoV exhibited increased viral N protein expression, RNA levels, and titers (Fig. 1M through O). Collectively, these results demonstrate that PEX13 exerts broad-spectrum antiviral activity against swine enteric coronaviruses, including PEDV, PDCoV, and TGEV.

### Virtual screening identifies Cyclo-C as a PEX13-dependent small-molecule inhibitor of PEDV

**Fig 2:**
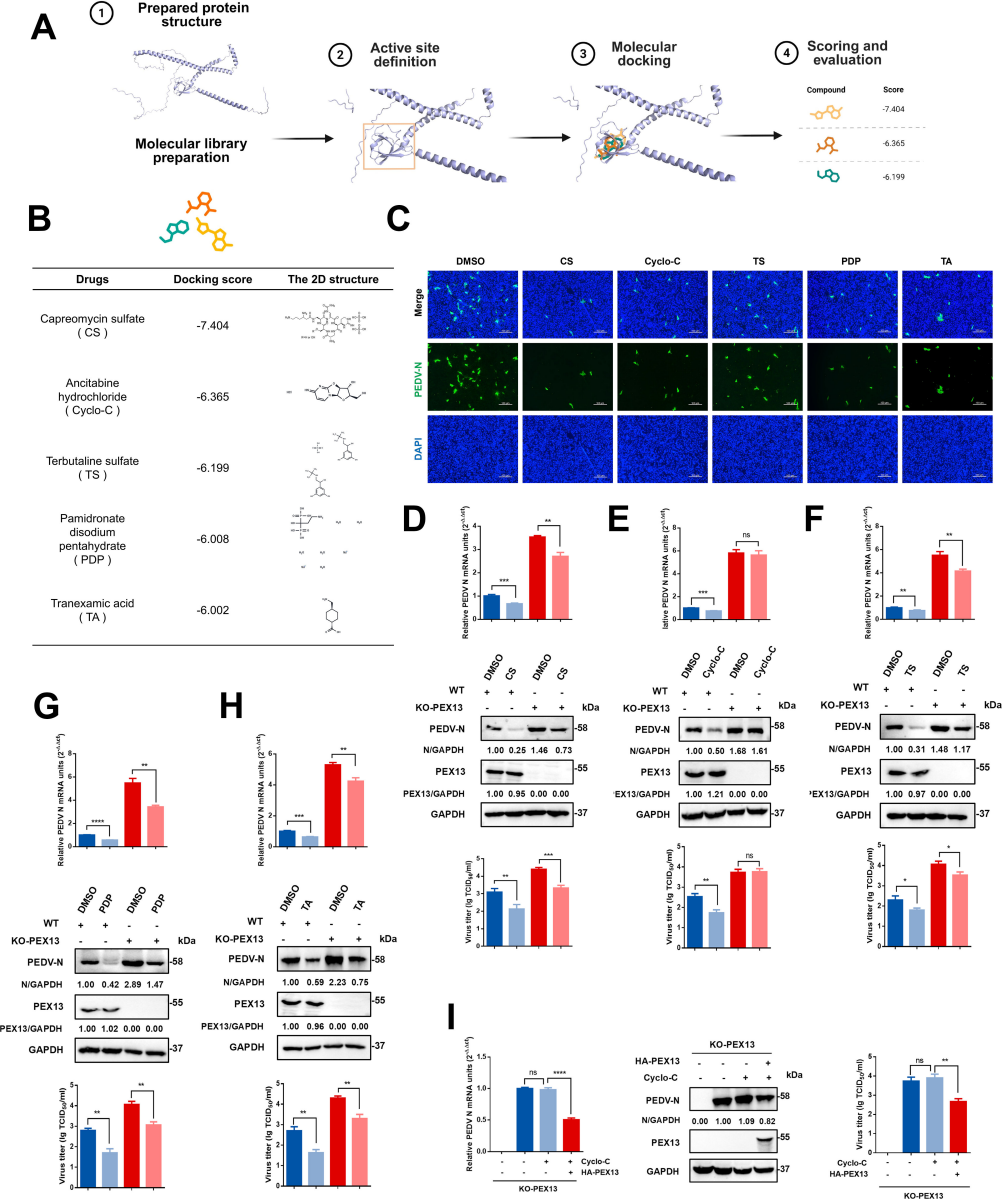
Identification of PEX13-targeting small-molecule inhibitors against PEDV. (**A**) Schematic representation of the structure-based virtual screening workflow used to identify candidate compounds targeting PEX13. (**B**) Molecular docking scores and two-dimensional chemical structures of the five compounds selected for experimental validation. (**C**) IFA detecting PEDV infection in IPEC-J2 cells treated with 10 μM of the indicated compounds. IPEC-J2 cells were pretreated with the test compounds for 2 h, then infected with PEDV (MOI = 5) for 2 h. The medium was then replaced with maintenance medium containing the corresponding compounds. Cells were harvested and fixed at 24 hpi. PEDV nucleocapsid (N) protein is shown in green. Bars = 100 µm. (**D–H**) Western blotting analysis, RT-qPCR, and TCID_50_ of PEDV infection in WT or PEX13-KO IPEC-J2 cells treated with 10 μM of the indicated compounds. Data are means ± SD from three independent experiments. *, *P* < 0.05; **, *P* < 0.01; ***, *P* < 0.001; ****, *P* < 0.0001; ns = not significant. (**I**) In IPEC-J2 PEX13-KO cells overexpressing HA-PEX13 or empty vector, after PEDV infection and treatment with Cyclo-C or DMSO-containing maintenance medium for 24 h, viral replication was quantified by Western blotting analysis of viral protein expression, RT-qPCR targeting PEDV N mRNA, and TCID_50_ assay. Data are means ± SD from three independent experiments. **, *P* < 0.01; ****, *P* < 0.0001; ns = not significant.

To identify small-molecule modulators that enhance the antiviral function of PEX13, we conducted structure-based virtual screening of compound libraries against the three-dimensional structure of PEX13 (Fig. 2A). Based on predicted binding energies and drug-like properties, five candidate compounds, capreomycin sulfate, Cyclo-C, terbutaline sulfate, pamidronate disodium pentahydrate, and tranexamic acid, were selected for experimental validation (Fig. 2B). Immunofluorescence assay (IFA) confirmed that all five compounds effectively suppressed PEDV infection (Fig. 2C).

To determine whether the antiviral effects of these compounds are mediated through PEX13, WT, and PEX13-KO IPEC-J2 cells were pretreated with 10 μM each compound for 2 h, followed by PEDV infection and maintained in compound-containing medium. At 24 hpi, cells were harvested and subjected to RT-qPCR, Western blotting, and virus titration. All five compounds significantly inhibited PEDV replication in WT cells. Notably, however, only Cyclo-C lost its antiviral activity in PEX13-KO cells (Fig. 2D through H), suggesting that Cyclo-C suppresses PEDV replication in a PEX13-dependent manner.

To further investigate whether the anti-PEDV effect of Cyclo-C depends on PEX13, a rescue experiment was performed by transfecting PEX13-KO cells with either the HA-PEX13 recombinant plasmid or the pCAGGS empty vector control plasmid. At 24 h post-transfection, cells were infected with PEDV and maintained in medium containing either Cyclo-C or DMSO (vehicle control). Samples were harvested at 24 hpi for analysis by Western blotting, RT-qPCR, and virus titration. The results demonstrated that in PEX13-KO cells, Cyclo-C treatment had no significant effect on PEDV infection compared to the DMSO control group. However, upon re-expression of HA-PEX13 (rescue), Cyclo-C treatment significantly inhibited PEDV infection. These findings were consistently observed across all three detection methods, as shown in Fig. 2I.

### Cyclo-C specifically binds to PEX13 and promotes its protein accumulation

**Fig 3:**
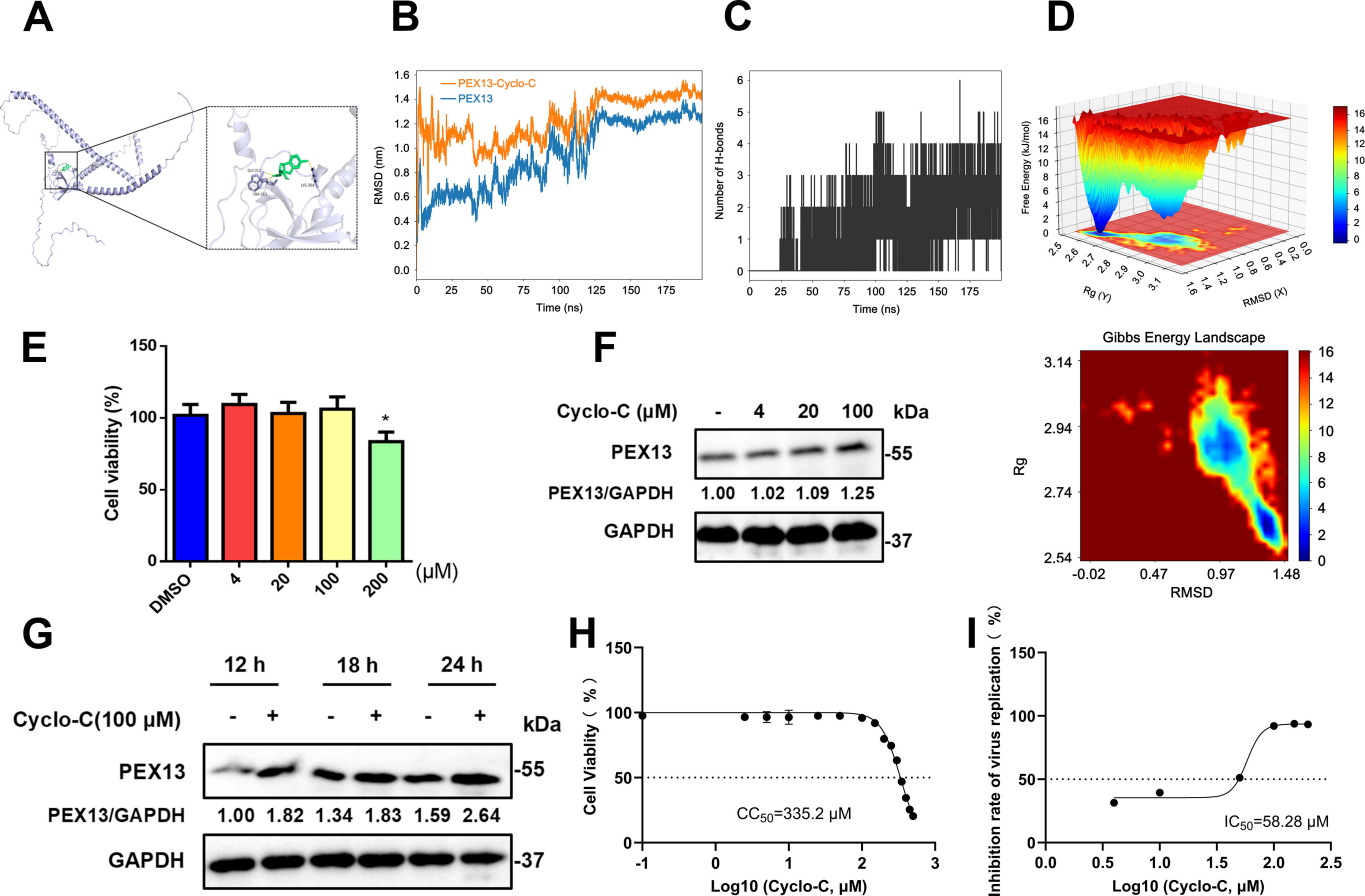
Cyclo-C directly binds to PEX13 and promotes its protein accumulation. (**A**) Molecular docking model depicting the predicted interaction between porcine PEX13 (purple) and Cyclo-C (green). Hydrogen bonds formed with residues LYS304, GLY312, and TRP313 are shown as dashed lines. (**B**) Root mean square deviation (RMSD) analysis of PEX13 alone (blue) and the PEX13–Cyclo-C complex (orange) during a 200-ns molecular dynamics (MD) simulation. (**C**) Number of hydrogen bonds formed between PEX13 and Cyclo-C throughout the 200-ns MD simulation. (**D**) Gibbs free energy landscape constructed from principal component analysis (PCA) of the MD trajectories. The low nm and radius of gyration (Rg) of 2.54–2.74 nm. (**E**) Cell viability determined by CCK-8 assay after 24-h treatment of IPEC-J2 cells with Cyclo-C. Data are means ± SD from three independent experiments. *, *P* < 0.05. (**F**) Western blotting analysis of PEX13 expression in IPEC-J2 cells treated with increasing concentrations of Cyclo-C for 24 h. (**G**) Time-dependent changes in PEX13 protein levels in IPEC-J2 cells treated with Cyclo-C (100 μM). (**H**) Determination of the 50% cytotoxic concentration (CC_50_) of Cyclo-C. CC_50_ was calculated from the viability curve. IPEC-J2 cells were treated with graded concentrations of Cyclo-C for 24 h. Cell viability was tested using an enhanced CCK-8 following the manufacturer’s instructions. The CC_50_ was calculated using GraphPad Prism 7.0 software. (**I**) Dose % inhibitory concentration (IC_50_) of Cyclo-C and treatment with increasing concentrations of Cyclo-C post. IPEC-J2 cells were infected with PEDV (MOI = 1) and treated with graded concentrations of Cyclo-C (4, 10, 50, 100, 150, and 200 μM). At 24 hpi, PEDV N protein expression was quantified by immunofluorescence, and fluorescence intensity was measured using ImageJ software. The percentage inhibition of PEDV replication at each concentration relative to the DMSO control was calculated, and the IC_50_ was determined by nonlinear regression analysis using GraphPad Prism 7.0.

To further validate the direct interaction between Cyclo-C and PEX13, molecular docking analysis was performed using AutoDock. Cyclo-C exhibited a favorable binding free energy of −6.365 kcal/mol with PEX13. Visualization of the docking pose revealed that Cyclo-C forms three hydrogen bonds with residues LYS304, GLY312, and TRP313 of PEX13 (Fig. 3A), suggesting a stable interaction between Cyclo-C and PEX13.

To assess the stability of the predicted binding conformation, we performed MD simulations of the PEX13–Cyclo-C complex over 200 ns. MD simulations apply Newtonian mechanics to model atomic motions in solvated systems and represent a robust computational approach for evaluating protein–drug interactions (38, 39). Complex stability was assessed using RMSD analysis. In the PEX13–Cyclo-C system, RMSD values converged below 1.4 nm and stabilized after approximately 125 ns, indicating the formation of a stable binding conformation (Fig. 3B). Hydrogen bond analysis was performed throughout the simulation to characterize intermolecular polar interactions (40). Cyclo-C consistently formed hydrogen bonds with residues within the PEX13-binding pocket, contributing to the overall stability of the complex (Fig. 3C). Furthermore, the Gibbs free energy landscape was constructed based on PCA of the MD trajectories (41). The resulting free energy surface revealed a distinct low-energy basin corresponding to the bound state of Cyclo-C with PEX13, with an RMSD of approximately 0.97–1.48 nm and a radius of gyration (Rg) of 2.54–2.74 nm, indicating a compact and stable complex conformation (Fig. 3D). Collectively, these computational analyses demonstrate that Cyclo-C binding stabilizes the structure of PEX13.

To establish appropriate experimental conditions for functional studies, we evaluated the cytotoxicity of Cyclo-C in IPEC-J2 cells using the CCK-8 assay. Cell viability assays identified 100 μM as the maximum non-cytotoxic concentration of Cyclo-C (Fig. 3E). We next investigated the effect of Cyclo-C on PEX13 protein expression. IPEC-J2 cells were treated with increasing concentrations of Cyclo-C for 24 h, and Western blotting analysis revealed that Cyclo-C upregulated PEX13 protein expression in a dose-dependent manner (Fig. 3F). Time-course experiments further demonstrated that treatment with 100 μM Cyclo-C significantly enhanced PEX13 protein accumulation over time (Fig. 3G). The CC_50_ of Cyclo-C in IPEC-J2 cells was determined to be 335.2 μM (Fig. 3H), while the IC_50_ against PEDV was 58.28 μM, indicating potent anti-PEDV activity with a favorable selectivity index (Fig. 3I).

### Cyclo-C primarily inhibits the replication stage of PEDV

**Fig 4:**
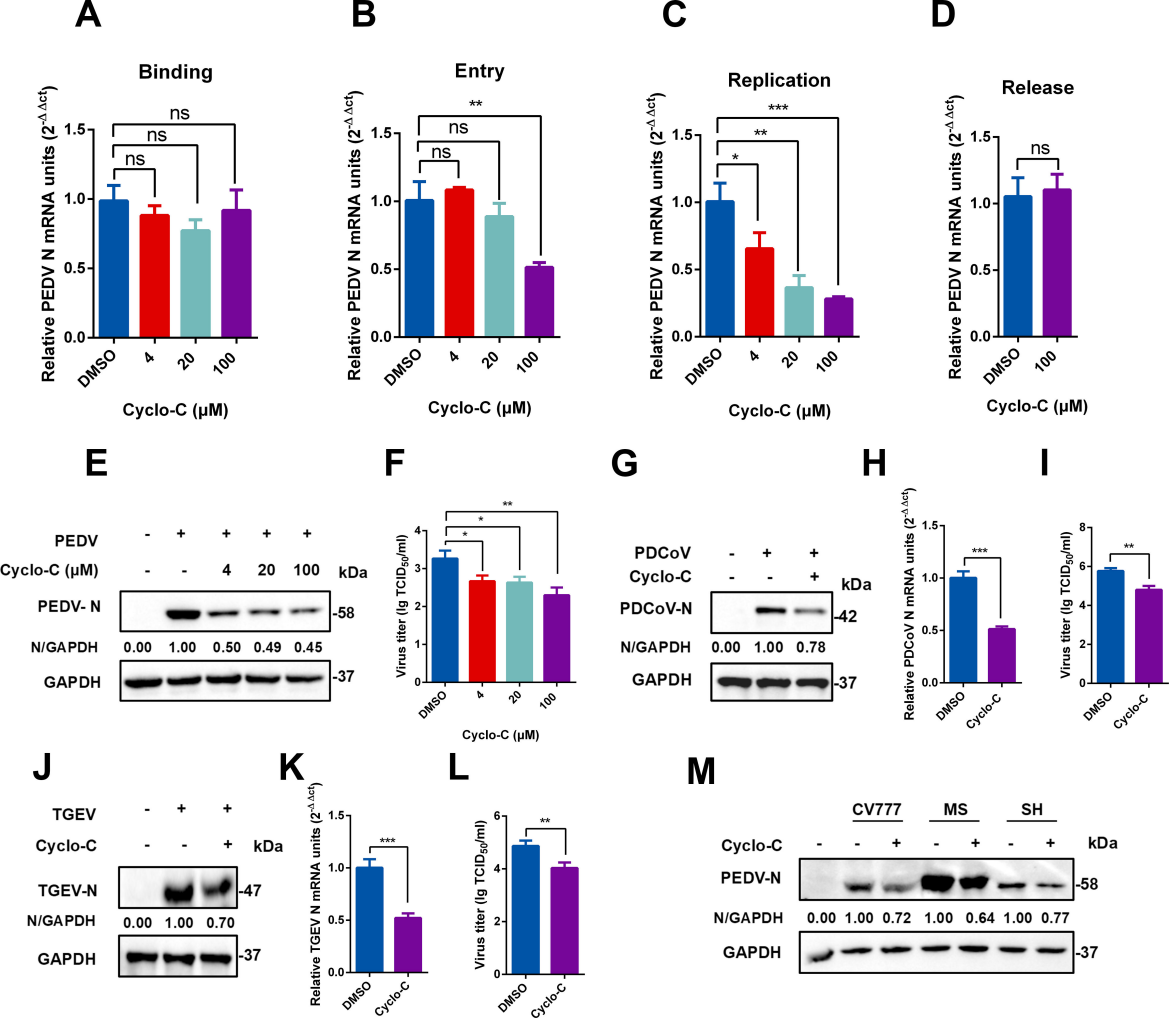
Cyclo-C primarily inhibits the replication stage of PEDV. (**A**) Virus binding assay. IPEC-J2 cells were pretreated with Cyclo-C for 2 h and infected with PEDV (MOI = 10) at 4°C for 1 h. Viral RNA levels were quantified by RT-qPCR. Data are means ± SD from three independent experiments. ns = not significant. (**B**) Virus entry assay. IPEC-J2 cells were pretreated with Cyclo-C and infected with PEDV (MOI = 10) at 37°C for 2 h, followed by RT-qPCR analysis. Data are means ± SD from three independent experiments. **, *P* < 0.01; ns = not significant. (**C**) Virus replication assay. IPEC-J2 cells were infected with PEDV (MOI = 1) for 2 h, then treated with Cyclo-C for 22 h. Viral RNA was measured by RT-qPCR. Data are means ± SD from three independent experiments. *, *P* < 0.05; **, *P* < 0.01; ***, *P* < 0.001. (**D**) Virus release assay. IPEC-J2 cells were infected with PEDV (MOI = 1) for 16 h, followed by Cyclo-C treatment for 8 h. Viral RNA was quantified by RT-qPCR. Data are means ± SD from three independent experiments. ns = not significant. (E–F) IPEC-J2 cells were infected with PEDV (MOI = 1). At 2 hpi, cells were treated with specified concentrations of Cyclo-C for 22 h. Viral replication was quantified by Western blotting analysis of viral protein expression (**E**) and TCID_50_ assay (**F**). Data are means ± SD from three independent experiments. *, *P* < 0.05; **, *P* < 0.01. (G–I) Cyclo-C inhibits PDCoV infection in IPEC-J2 cells. IPEC-J2 cells were infected with PDCoV at an MOI of 1. At 2 hpi, cells were treated with 100 μM of Cyclo-C for 22 h. Viral replication in IPEC-J2 cells was quantified by Western blotting (**G**), RT-qPCR (**H**), and TCID_50_ assay (**I**). Data are means ± SD from three independent experiments. **, *P* < 0.01; ***, *P* < 0.001. (**J–L**) Cyclo-C inhibits TGEV infection in ST cells. ST cells infected with TGEV were treated with Cyclo-C (100 μM) for 22 h. At 24 hpi, samples were collected for Western blotting (**J**), RT-qPCR (**K**), and TCID_50_ assay (**L**). Data are means ± SD from three independent experiments. **, *P* < 0.01; ***, *P* < 0.001. (**M**) Western blot of N protein in cells infected with different PEDV genotypes and treated with 100 μM Cyclo-C or DMSO at 24 hpi.

Viral infection typically proceeds through binding, entry, replication, and release. To determine which stage of PEDV infection is affected by Cyclo-C, we systematically examined its effects during viral infection. Pretreatment of IPEC-J2 cells with Cyclo-C did not significantly affect PEDV binding (Fig. 4A). Viral entry was only modestly inhibited at high concentrations of Cyclo-C (Fig. 4B). To assess the effect on viral replication, IPEC-J2 cells were infected with PEDV for 2 h, followed by replacement with maintenance medium containing increasing concentrations of Cyclo-C. Viral RNA levels were measured at 24 hpi by RT-qPCR. Cyclo-C markedly inhibited PEDV replication in a dose-dependent manner (Fig. 4C). To evaluate viral release, PEDV-infected cells were treated with 100 μM Cyclo-C at 18 hpi, and viral RNA in the supernatant was quantified at 24 hpi. Cyclo-C did not significantly affect viral release compared with the DMSO control (Fig. 4D).

Moreover, Western blotting and 50% tissue culture infective dose (TCID_50_) assays were performed to further confirm the inhibitory effect of Cyclo-C on PEDV replication. IPEC-J2 cells were infected with PEDV for 2 h, followed by replacement with maintenance medium containing graded concentrations of Cyclo-C. The samples were harvested at PEDV 24 hpi. Collectively, these results establish Cyclo-C as a replication-stage-specific inhibitor of PEDV (Fig. 4E and F).

To determine whether Cyclo-C exhibits antiviral activity against other porcine coronaviruses, we examined its effects on PDCoV and TGEV. IPEC-J2 and ST cells were infected with PDCoV or TGEV, respectively, for 2 h and subsequently cultured in maintenance medium containing 100 μM Cyclo-C. Cells were harvested at 24 hpi. The results showed that Cyclo-C significantly inhibited the replication of both PDCoV and TGEV (Fig. 4G through L). These findings indicate that Cyclo-C possesses broad antiviral activity against multiple swine coronaviruses.

In addition, to evaluate the antiviral efficacy of Cyclo-C against various PEDV genotypes, the classic G1 strain (CV777) and G2b variant strains (MS and SH) were selected for study. IPEC-J2 cells were infected with these strains and subsequently treated with Cyclo-C or DMSO. Samples were harvested at 24 hpi for analysis by Western blotting. The results demonstrated that Cyclo-C exerted similar inhibitory effects on the tested variant strains (MS and SH) as well as the classic CV777 strain (Fig. 4M). These findings indicate that Cyclo-C possesses broad-spectrum antiviral activity against multiple PEDV genotypes.

### Cyclo-C disrupts the PEX13–NSP8 interaction and counteracts NSP8-mediated PEX13 degradation

**Fig 5:**
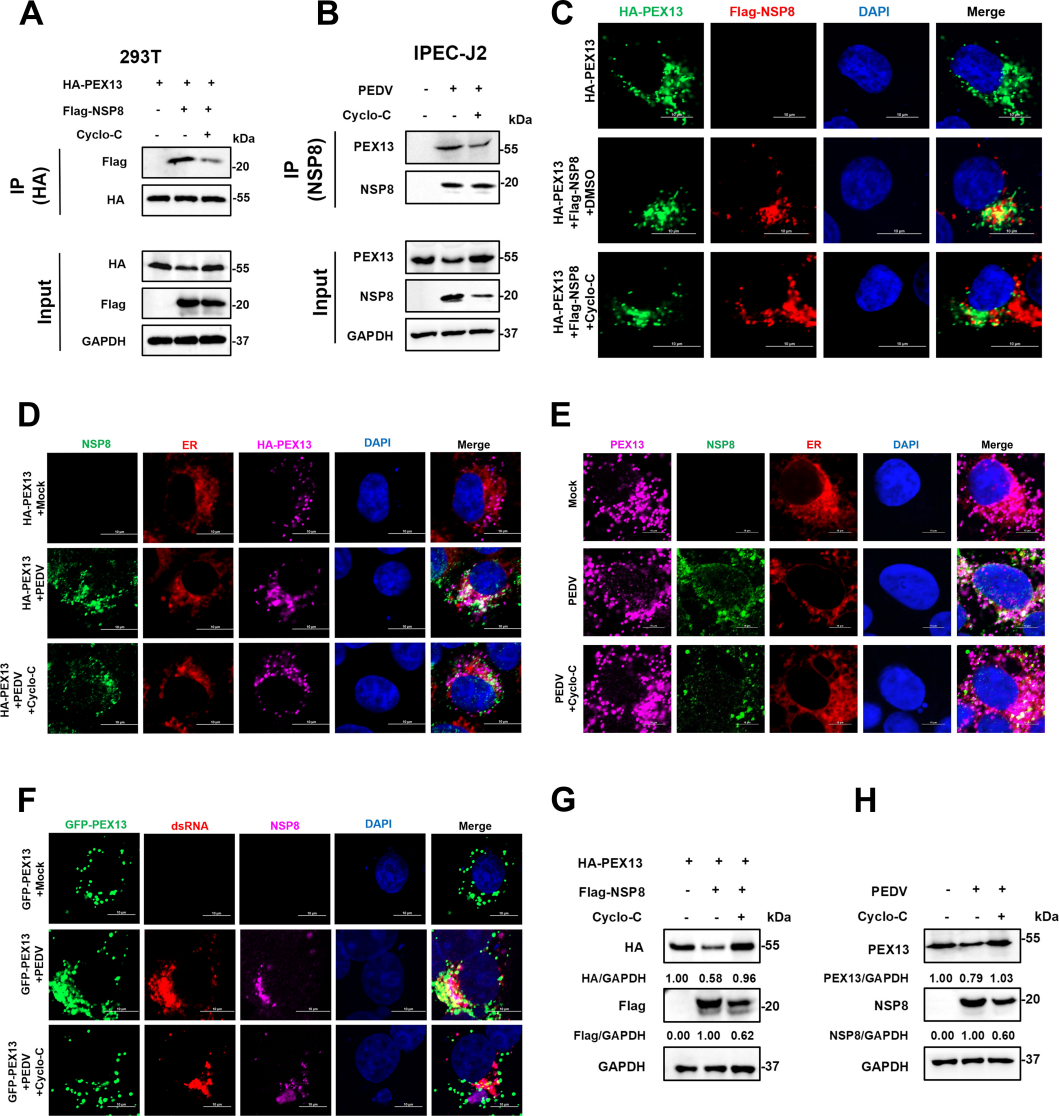
Cyclo-C disrupts the PEX13–NSP8 interaction and counteracts NSP8-mediated PEX13 degradation. (**A**) Cyclo-C inhibits the interaction between PEX13 and NSP8. 293T cells seeded in 60-mm dishes were transfected with either Flag-NSP8 (8 μg) or empty vector pxj41 (8 μg) for 6 h, followed by medium replacement. After an additional 6 h, cells were secondarily transfected with either HA-PEX13 (4 μg) or pxj41 (4 μg) for another 6 h, then switched to medium containing either DMSO or Cyclo-C. Cells were processed for immunoprecipitation (IP) using anti-HA magnetic beads. Both whole-cell lysates (input) and immunoprecipitated proteins were analyzed by immunoblotting with antibodies against Flag-tag, HA-tag, and GAPDH. (**B**) IPEC-J2 cells seeded in 60-mm dishes were infected with PEDV, then replaced with maintenance medium containing either Cyclo-C or DMSO. At 12 hpi, cells were subjected to immunoprecipitation using anti-NSP8 antibody. Both input proteins and immunoprecipitated proteins were analyzed by Western blot with antibodies against NSP8, PEX13, and GAPDH. (**C**) Cyclo-C reduced cytoplasmic colocalization of NSP8 and PEX13. 293T cells were sequentially transfected with Flag-NSP8 (4 μg) together with either an empty vector or HA-PEX13 (2 μg). The medium was then replaced with DMSO- or Cyclo-C-containing medium. At 36 hpt, cells were stained with anti-HA (green) and anti-Flag (red) antibodies for confocal microscopy analysis. Nuclei were counterstained with DAPI (blue). Scale bar = 10 µm. (**D**) IPEC-J2 cells transfected with HA-PEX13 and infected with PEDV (MOI = 10), the medium was then replaced with DMSO- or Cyclo-C-containing medium. At 12 hpi, the cells were fixed and probed with mouse anti-NSP8 antibody (green), ER-Tracker Red (red), rabbit anti-HA antibody (purple), and then observed under a confocal microscope. Bars = 10 µm. (**E**) IPEC-J2 cells infected with PEDV (MOI = 10) were treated with maintenance medium containing Cyclo-C or DMSO. At 12 hpi, cells were fixed and subjected to immunofluorescence staining using mouse anti-NSP8 antibody (green), ER-Tracker Red (red), and rabbit anti-PEX13 antibody (purple). Images were acquired with a confocal microscope. Bars = 10 µm. (**F**) Confocal microscopy showing decreased association of NSP8 with GFP-PEX13 and dsRNA in Cyclo-C-treated cells. IPEC-J2 cells transfected with GFP-PEX13 and infected with PEDV (MOI = 10), the medium was then replaced with DMSO- or Cyclo-C-containing medium. At 12 hpi, the cells were fixed and probed with mouse anti-NSP8 antibody (purple) and rabbit anti-dsRNA antibody (red), then observed under a confocal microscope. Scale bar = 10 µm. (**G**) Cyclo-C alleviates NSP8-mediated degradation of PEX13 in PEX13-OE IPEC-J2 cells. In the PEX13-OE IPEC-J2 cells, after transfection with Flag-NSP8 or the pXJ41 empty vector, the medium was replaced with Cyclo-C or DMSO. Samples were harvested at 24 hpi, and Western blotting was performed using anti-HA, anti-Flag, and anti-GAPDH antibodies. (**H**) Cyclo-C attenuates endogenous NSP8-mediated degradation of PEX13 in IPEC-J2 cells. Cells seeded in 12-well plates were infected with PEDV and treated with maintenance medium containing Cyclo-C or DMSO. At 24 hpi, cells were harvested for immunoblotting analysis using antibodies against PEX13, NSP8, and GAPDH.

We previously reported that PEDV NSP8 interacts with PEX13, promoting its degradation and subsequent peroxisome depletion, thereby suppressing the type III interferon (IFN-III) response and facilitating viral replication (26). Given that PEDV exploits the NSP8–PEX13–pexophagy axis to evade IFN-III-mediated antiviral immunity, we sought to investigate whether Cyclo-C modulates this critical virus–host interaction. To test this, 293T cells were co-transfected with Flag-NSP8 and HA-PEX13 and treated with either DMSO or Cyclo-C. Co-immunoprecipitation (Co-IP) assays revealed that Cyclo-C treatment markedly reduced the interaction between Flag-NSP8 and HA-PEX13 (Fig. 5A). To evaluate whether Cyclo-C inhibits the PEX13-NSP8 complex endogenously during PEDV infection, IPEC-J2 cells infected with PEDV (MOI = 10) and treated with either Cyclo-C or DMSO were subjected to Co-IP assays using an anti-NSP8 antibody. As shown in Fig. 5B, Cyclo-C treatment significantly suppressed the endogenous interaction between NSP8 and PEX13 compared to the control group. This finding was corroborated by confocal microscopy, which showed decreased colocalization of the two proteins upon Cyclo-C treatment (Fig. 5C).

As a coronavirus, PEDV utilizes NSP8 as a key component of its viral replication complex, composed of NSP7, NSP8, NSP12, and double-stranded RNA (dsRNA), which forms the core of the Replication–Transcription complex (RTC). NSP8 can bind RNA templates to initiate the synthesis of complementary oligonucleotides and exhibits secondary RNA-dependent RNA polymerase (RdRp) activity (42, 43). Our previous work demonstrated that following PEDV infection, NSP8 recruits PEX13 to the endoplasmic reticulum (ER). To determine whether Cyclo-C affects NSP8-mediated relocalization of PEX13, we performed confocal microscopy to visualize the spatial relationship among PEX13, NSP8, and the ER. The results showed that Cyclo-C treatment reduced the co-localization of NSP8 with either endogenous or exogenous PEX13 on the ER, preventing the recruitment of both exogenous and endogenous PEX13 to the ER (Fig. 5D and E). To further assess whether Cyclo-C interferes with the association between PEX13 and the RTC complex, IPEC-J2 cells expressing GFP-PEX13 were infected with PEDV and cultured in the presence or absence of Cyclo-C. At 12 hpi, confocal microscopy revealed that Cyclo-C treatment reduced the colocalization of PEX13 with both NSP8 and dsRNA, indicating impaired recruitment of PEX13 to sites of viral RNA synthesis (Fig. 5F). We next examined whether Cyclo-C could counteract NSP8-mediated PEX13 degradation. IPEC-J2 cells overexpressing HA-PEX13 were transfected with Flag-NSP8 and subsequently treated with DMSO or Cyclo-C for 24 h. Western blotting analysis demonstrated that Cyclo-C treatment significantly restored HA-PEX13 protein levels compared to the DMSO-treated control (Fig. 5G). Similarly, Cyclo-C alleviated the inhibitory effect of PEDV on endogenous PEX13 (Fig. 5H). Notably, given that Cyclo-C directly upregulates PEX13 expression (Fig. 3F and G), the observed restoration of PEX13 protein levels in the presence of NSP8 could, in principle, reflect either increased synthesis or reduced degradation. The disrupted NSP8–PEX13 interaction demonstrated by co-immunoprecipitation and colocalization (Fig. 5A through C) supports the latter mechanism. Collectively, these findings indicate that Cyclo-C disrupts the NSP8–PEX13 interaction, alters PEX13 subcellular localization, prevents its recruitment to viral replication complexes, and ultimately blocks NSP8-mediated PEX13 degradation.

### Cyclo-C inhibits PEDV infection by blocking pexophagy

**Fig 6:**
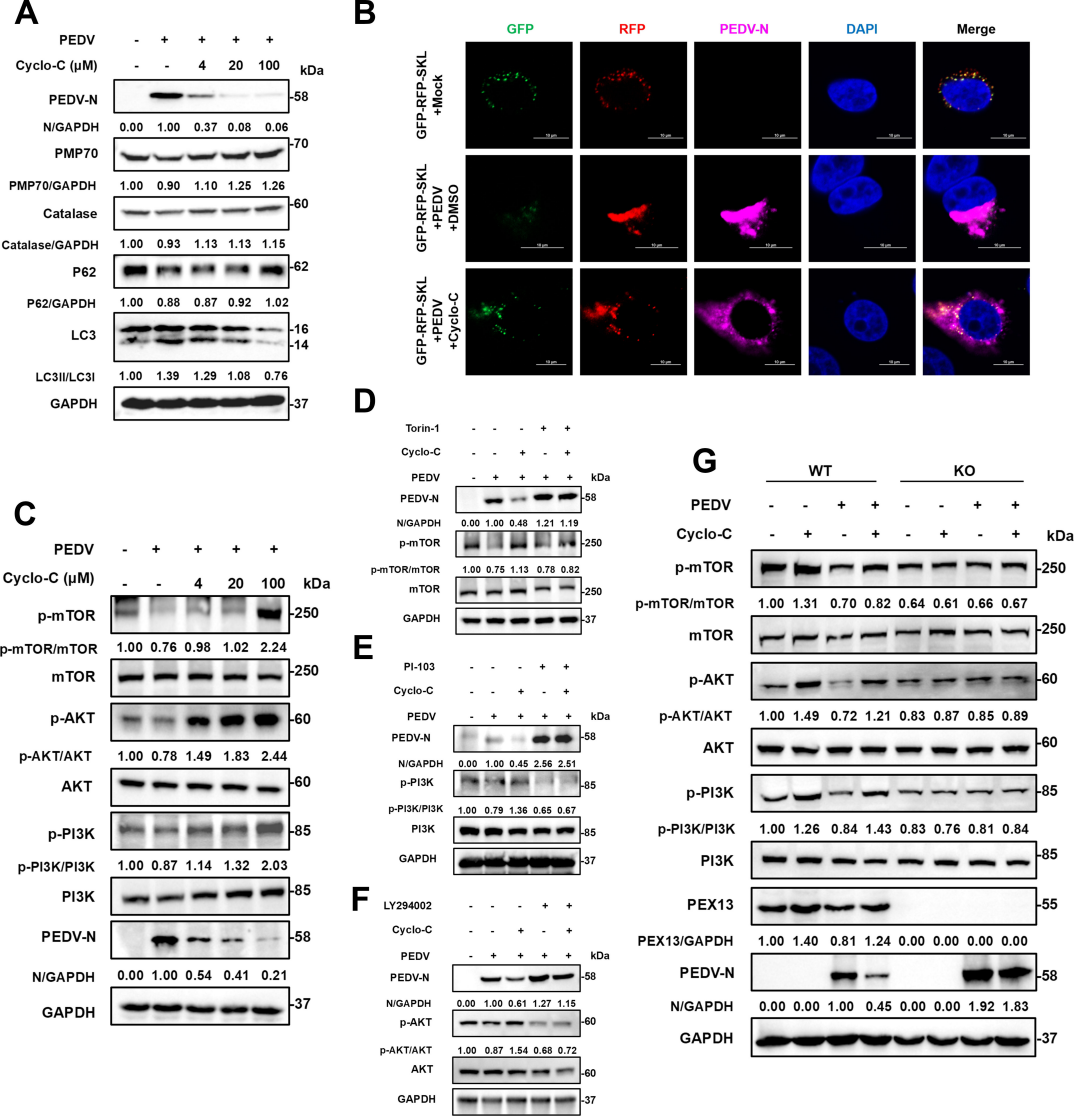
Cyclo-C inhibits PEDV infection by blocking pexophagy. (**A**) Cyclo-C alleviates PEDV-induced pexophagy in a dose-dependent manner. IPEC-J2 cells were seeded in six-well plates and then infected with PEDV. Subsequently, the medium was replaced with a maintenance solution containing the corresponding concentration of Cyclo-C. At 24 hpi, the cells were collected for Western blotting analysis. (**B**) Confocal microscopy of GFP-RFP-SKL reporter showing that Cyclo-C suppresses PEDV-induced pexophagy. IPEC-J2 cells were transfected with GFP-RFP-SKL for 24 h, then mock-infected or infected with PEDV. Cells were treated with DMSO or Cyclo-C (100 μM) at 2 hpi with PEDV. After another 22 hpi, cells were incubated with anti-PEDV N antibody for immunostaining. (**C**) Cyclo-C restores phosphorylation of PI3K, AKT, and mTOR in a dose-dependent manner in PEDV-infected cells. IPEC-J2 cells were seeded in 12-well plates and infected with PEDV, followed by replacement with maintenance medium containing corresponding concentrations of Cyclo-C. At 24 hpi, cells were harvested for Western blotting analysis using antibodies against PI3K, phosphorylated PI3K (p-PI3K), AKT, phosphorylated AKT (p-AKT), mTOR, phosphorylated mTOR (p-mTOR), and GAPDH. (D–F) Cyclo-C suppresses PEDV replication through the activation of PI3K/AKT/mTOR signaling pathways. IPEC-J2 cells were infected with PEDV for 2 h, followed by treatment with Cyclo-C and/or mTOR inhibitor (Torin-1) (**D**), PI3K inhibitor (PI-103) (**E**), and AKT inhibitor (LY294002) (**F**), for 22 h. Cells were then harvested for Western blotting analysis. (**G**) Cyclo-C targets PEX13 to activate the PI3K/AKT/mTOR signaling pathway. IPEC-J2 WT and PEX13-KO cells were infected with PEDV for 2 h, followed by treatment with Cyclo-C for 22 h. Cells were then harvested for Western blotting analysis using antibodies against PI3K, p-PI3K, AKT, p-AKT, mTOR, p-mTOR, PEX13, PEDV-N, and GAPDH.

We previously demonstrated that PEDV infection promotes pexophagy by downregulating PEX13 expression, leading to peroxisome depletion (26). Peroxisomes can be monitored using PMP70 (a peroxisomal membrane protein) and catalase (a peroxisomal matrix enzyme) as established markers (44). To investigate whether Cyclo-C modulates PEDV-induced pexophagy, IPEC-J2 cells were infected with PEDV, treated with increasing concentrations of Cyclo-C, and harvested at 24 hpi for immunoblot analysis. PEDV infection markedly reduced the protein levels of both PMP70 and catalase compared to mock-infected controls. Notably, Cyclo-C treatment restored the expression of both peroxisomal markers in a dose-dependent manner. Furthermore, Cyclo-C reversed PEDV-induced p62 depletion and attenuated the elevation of the LC3-II/LC3-I ratio, indicating suppression of autophagic flux (Fig. 6A). To directly visualize the effect of Cyclo-C on pexophagosome formation, cells were transfected with the RFP-GFP-SKL dual-fluorescence reporter plasmid, which targets peroxisomes via the PTS1 signal sequence. In this system, GFP fluorescence is quenched in acidic lysosomal compartments while RFP remains stable, enabling discrimination between cytosolic peroxisomes (GFP^+^/RFP^+^) and those delivered to lysosomes for degradation (GFP^−^/RFP^+^). Confocal microscopy revealed that PEDV infection significantly reduced GFP/RFP colocalization, indicative of enhanced pexophagic flux. Cyclo-C treatment effectively restored the GFP/RFP signal (Fig. 6B), confirming that Cyclo-C suppresses PEDV-induced pexophagy.

The PI3K/AKT/mTOR signaling axis is a master regulator of autophagy, and inhibition of mTOR activity has been shown to promote pexophagy (45, 46). To elucidate the molecular mechanism by which Cyclo-C inhibits pexophagy, we examined the phosphorylation status of PI3K, AKT, and mTOR in Cyclo-C-treated cells. PEDV infection markedly suppressed the ratios of phosphorylated to total PI3K, AKT, and mTOR. In contrast, Cyclo-C treatment dose-dependently restored phosphorylation of all three signaling components (Fig. 6C), suggesting that activation of the PI3K/AKT/mTOR pathway is a key feature of Cyclo-C-mediated suppression of pexophagy. To functionally validate this mechanism, we employed pharmacological inhibitors of PI3K (PI-103), AKT (LY294002), and mTOR (Torin-1) in PEDV-infected cells treated with Cyclo-C. Inhibition of each signaling component reversed the antiviral effect of Cyclo-C, as evidenced by restored PEDV replication (Fig. 6D through F). Collectively, these findings indicate that Cyclo-C exerts its antiviral activity by activating the PI3K/AKT/mTOR pathway, thereby inhibiting PEDV-induced pexophagy and preserving peroxisome integrity.

To investigate whether PEX13 is required for Cyclo-C-mediated activation of the PI3K/AKT/mTOR pathway, WT cells and PEX13 knockout (PEX13-KO) cells were infected with PEDV and treated with Cyclo-C or DMSO. The phosphorylation levels of PI3K, AKT, and mTOR were then examined. The results showed that, in WT cells, Cyclo-C treatment significantly increased the phosphorylation levels of PI3K/AKT/mTOR compared with the DMSO group, whereas PEDV infection markedly suppressed phosphorylation of this pathway. Notably, co-treatment with PEDV and Cyclo-C restored the phosphorylation levels of the PI3K/AKT/mTOR pathway. In PEX13-KO cells, however, neither Cyclo-C nor PEDV treatment altered the phosphorylation levels of PI3K/AKT/mTOR compared with the control group (Fig. 6G). These findings indicate that Cyclo-C regulates the phosphorylation of the PI3K/AKT/mTOR pathway in a PEX13-dependent manner.

### Cyclo-C restores peroxisomal MAVS and potentiates IFN-III responses via PI3K/AKT/mTOR-mediated suppression of pexophagy

**Fig 7:**
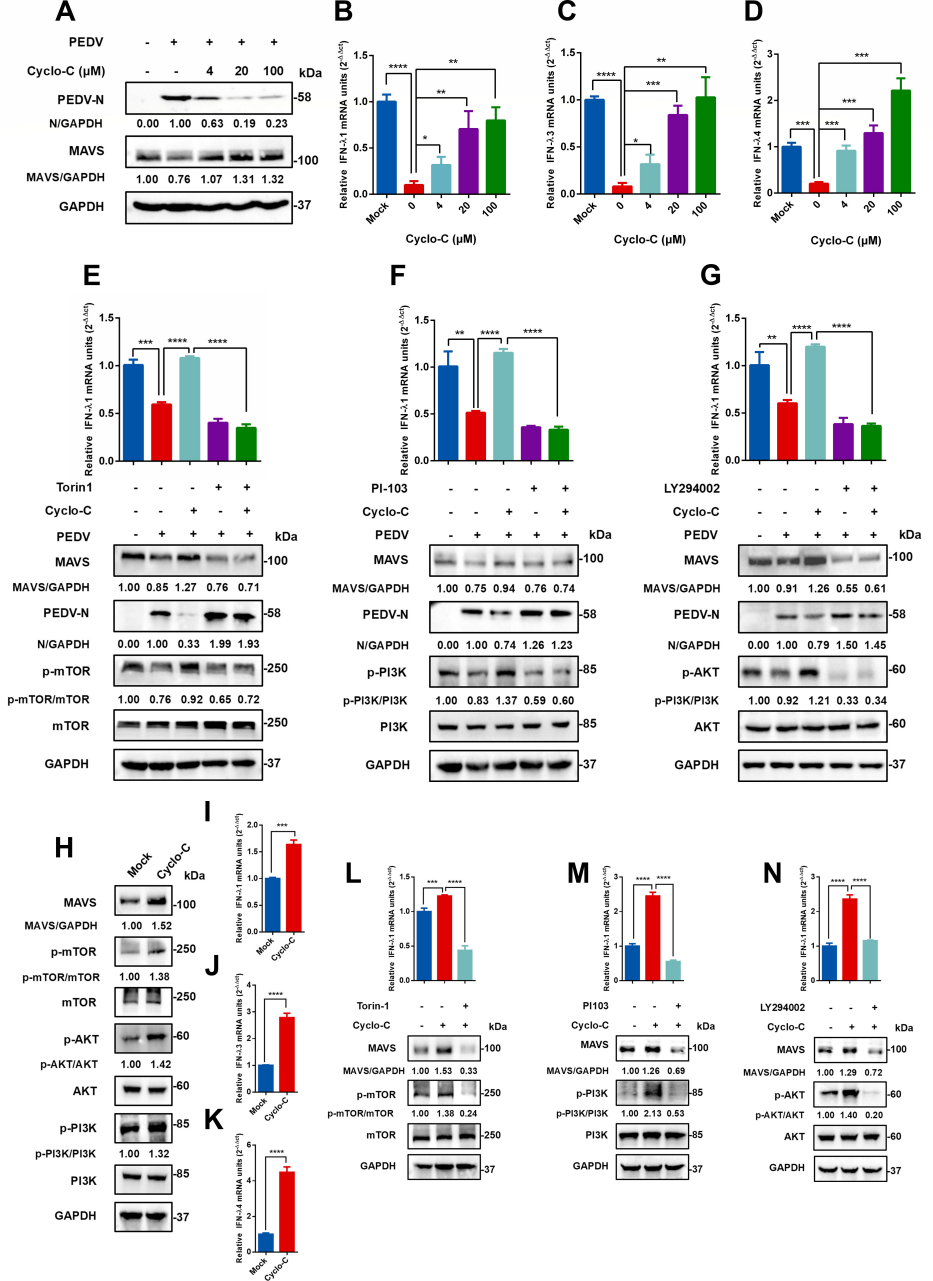
PI3K/AKT/mTOR-dependent inhibition of pexophagy by Cyclo-C sustains MAVS expression and IFN-III production. (**A**) Cyclo-C enhances MAVS protein levels following PEDV infection. IPEC-J2 cells were seeded in 12-well plates and then infected with PEDV. Subsequently, the medium was replaced with a maintenance medium containing the corresponding concentration of Cyclo-C. At 24 hpi, the cells were collected for Western blotting analysis. (**B–D**) Cyclo-C promotes the production of IFN-III (IFN-λ1, IFN-λ3, and IFN-λ4). IPEC-J2 cells were seeded in 12-well plates and then infected with PEDV. Subsequently, the medium was replaced with a maintenance medium containing the corresponding concentration of Cyclo-C. At 24 hpi, the cells were collected for RT-qPCR analysis. Data are means ± SD from three independent experiments. *, *P* < 0.05; **, *P* < 0.01; ***, *P* < 0.001. (**E–G**) Cyclo-C elevates MAVS protein levels via the mTOR/PI3K/AKT pathway in PEDV-infected cells. IPEC-J2 cells were pretreated with mTOR, PI3K, or AKT inhibitors, infected with PEDV, and then treated with maintenance medium containing 100 μM Cyclo-C at 1 hpi. Cells were harvested at 24 hpi. Western blotting was used to detect the protein levels of MAVS, PEDV-N, p-mTOR, mTOR, p-PI3K, PI3K, AKT, p-AKT, and GAPDH, and RT-qPCR was used to detect IFN-λ1 levels. Data are means ± SD from three independent experiments. **, *P* < 0.01; ***, *P* < 0.001; ****, *P* < 0.0001. (**H**) Cyclo-C elevates MAVS and the phosphorylation of PI3K, AKT, and mTOR protein levels in IPEC-J2 cells. IPEC-J2 cells were treated with maintenance medium containing 100 μM Cyclo-C for 24 h. Western blotting was used to detect the protein levels of MAVS, PEDV-N, p-mTOR, mTOR, p-PI3K, PI3K, AKT, p-AKT, and GAPDH. (**I–K**) Cyclo-C increased the mRNA levels of IFN-III in IPEC-J2 cells. IPEC-J2 cells were treated with maintenance medium containing 100 μM Cyclo-C for 24 h. RT-qPCR was used to detect the levels of IFN-λ1 (**I**), IFN-λ3 (**J**), and IFN-λ4 (**K**). Data are means ± SD from three independent experiments. ***, *P* < 0.001; ****, *P* < 0.0001. (**L–N**) Cyclo-C elevates MAVS protein levels via the mTOR/PI3K/AKT pathway in IPEC-J2 cells. IPEC-J2 cells were pretreated with mTOR, PI3K, or AKT inhibitors and 100 μM Cyclo-C for 24 h. Western blotting was used to detect the protein levels of MAVS, PEDV-N, p-mTOR, mTOR, p-PI3K, PI3K, AKT, p-AKT, and GAPDH, and RT-qPCR was used to detect IFN-λ1 levels. Data are means ± SD from three independent experiments. ***, *P* < 0.001; ****, *P* < 0.0001.

Pexophagy serves as the primary pathway for peroxisome turnover (47, 48). In intestinal epithelial cells, antiviral signaling mediated by IFN-III critically depends on MAVS localized to peroxisomes (49). Given our findings that Cyclo-C suppresses PEDV-induced pexophagy, we next investigated whether this effect translates into preservation of peroxisomal MAVS and enhanced IFN-III responses. To assess the impact of Cyclo-C on MAVS protein stability, IPEC-J2 cells were infected with PEDV and treated with increasing concentrations of Cyclo-C. Cell lysates were harvested at 24 hpi for immunoblot analysis. PEDV infection markedly reduced MAVS protein levels compared to mock-infected controls, whereas Cyclo-C treatment restored MAVS expression in a dose-dependent manner (Fig. 7A). We next examined whether Cyclo-C influences IFN-III production downstream of peroxisomal MAVS signaling. IPEC-J2 cells were infected with PEDV, treated with graded concentrations of Cyclo-C, and harvested at 24 hpi for RT-qPCR analysis of IFN-λ1, IFN-λ3, and IFN-λ4 mRNA levels. PEDV infection significantly suppressed the expression of all three IFN-λ subtypes. In contrast, Cyclo-C treatment dose-dependently alleviated this suppression (Fig. 7B through D). Collectively, these results indicate that Cyclo-C preserves peroxisomal MAVS and enhances IFN-III expression, thereby contributing to the restriction of PEDV infection.

To determine whether the Cyclo-C-mediated increase in MAVS protein and IFN-III expression requires activation of the PI3K/AKT/mTOR pathway, we performed pharmacological inhibition experiments. IPEC-J2 cells were infected with PEDV and treated with Cyclo-C in the presence or absence of pathway-specific inhibitors (PI-103 for PI3K, LY294002 for AKT, or Torin-1 for mTOR). MAVS protein levels and IFN-λ1 mRNA expression were assessed by Western blotting and RT-qPCR, respectively. Compared to Cyclo-C treatment alone, co-treatment with each pathway inhibitor significantly reduced both MAVS protein levels and IFN-λ1 expression, indicating that blockade of the PI3K/AKT/mTOR pathway abrogated the effects of Cyclo-C (Fig. 7E through G). To verify the direct regulatory effect of Cyclo-C on IFN-III and rule out the possibility that the increase in IFN-III levels after Cyclo-C treatment is an indirect result of reduced viral replication, IPEC-J2 cells were treated with Cyclo-C or DMSO for 24 h before sample collection. Western blotting was employed to detect the protein levels of MAVS and the phosphorylated forms of PI3K/AKT/mTOR, while RT-qPCR was used to measure the mRNA levels of IFN-λ1, IFN-λ3, and IFN-λ4. The results indicated that Cyclo-C significantly enhanced the protein levels of MAVS and the phosphorylated PI3K/AKT/mTOR (Fig. 7H). Cyclo-C also significantly upregulated the mRNA levels of IFN-λ1 (Fig. 7I), IFN-λ3 (Fig. 7J), and IFN-λ4 (Fig. 7K). Similarly, under PEDV-uninfected conditions, we performed pharmacological inhibition experiments. IPEC-J2 cells were treated with Cyclo-C in the presence or absence of pathway-specific inhibitors, including PI-103 for PI3K, LY294002 for AKT, and Torin-1 for mTOR. MAVS protein levels and IFN-λ1 mRNA expression were evaluated by Western blotting and RT-qPCR, respectively. Compared with Cyclo-C treatment alone, co-treatment with each pathway inhibitor significantly reduced MAVS protein levels and IFN-λ1 expression (Fig. 7L through N), indicating that blockade of the PI3K/AKT/mTOR pathway abolished Cyclo-C-mediated enhancement of MAVS expression and IFN-III responses. These findings demonstrate that Cyclo-C suppresses pexophagy through activation of the PI3K/AKT/mTOR pathway, thereby preserving MAVS stability and sustaining IFN-III production.

### Cyclo-C exhibits dose-dependent antiviral efficacy and mitigates intestinal pathology in PEDV-infected neonatal piglets

**Fig 8:**
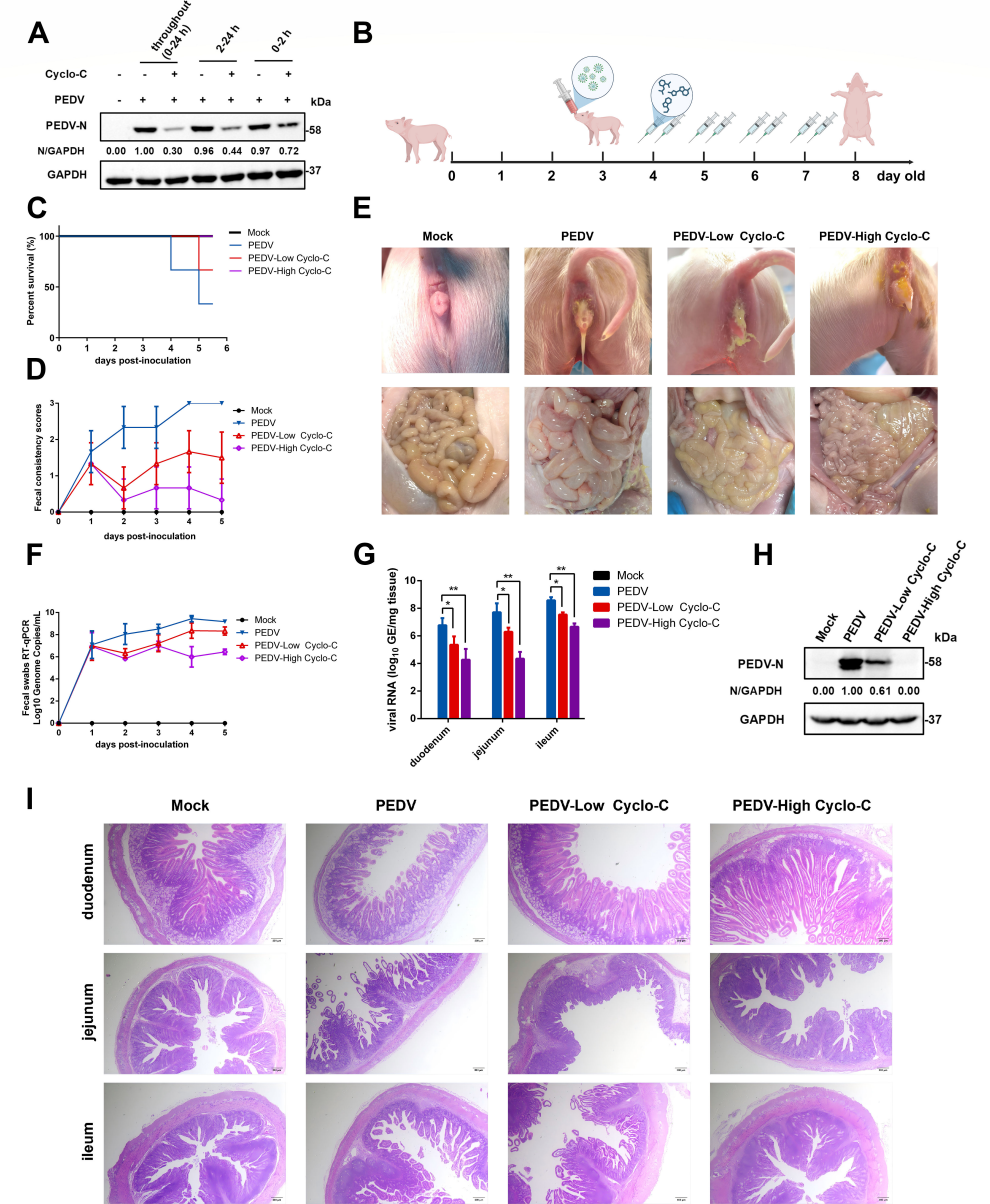
Therapeutic administration of Cyclo-C attenuates PEDV pathogenesis and mortality in neonatal piglets. (**A**) Time-of-addition assay showing that Cyclo-C primarily inhibits the replication stage of PEDV. IPEC-J2 cells were infected with PEDV, and Cyclo-C was added at different time points post-infection. Samples were collected at 24 hpi and analyzed by Western blotting to detect changes in PEDV N protein levels. (**B**) The schematic diagram of the animal experiment. (**C**) Therapeutic efficacy of Cyclo-C against PEDV infection in neonatal piglets. Three-day-old piglets were orally challenged with 2 × 10⁶ PFU of PEDV. Starting at 1 day post-infection (dpi), piglets received twice-daily treatments of Cyclo-C at a low dose (1.35 mg/kg) or a high dose (4 mg/kg). Survival rates were monitored and recorded daily. (**D**) Daily assessment of fecal consistency in PEDV-infected piglets. Fecal consistency was scored daily using the following scale: 0, solid; 1, pasty; 2, semi-liquid; and 3, liquid. (**E**) Representative clinical signs and gross pathological examination of piglets from different experimental groups. (**F**) Detection of viral RNA in fecal samples. Daily rectal swabs were collected from piglets and analyzed for PEDV RNA levels by RT-qPCR. (**G**) Viral RNA loads in small intestinal tissues. At 5 days post-challenge, small intestinal tissues were collected, and viral RNA copies per gram of tissue were quantified by RT-qPCR. Data are means ± SD from three independent experiments. *, *P* < 0.05; **, *P* < 0.01. (**H**) Levels of PEDV N protein in intestinal tissues. PEDV N protein levels per gram of colon tissue were measured in infected piglets at 5 days post-challenge. (**I**) Histopathological examination of small intestinal tissues from piglets in different treatment groups. Piglets were challenged with PEDV and euthanized 4 days after Cyclo-C treatment. Tissue sections from the duodenum, jejunum, and ileum were collected and subjected to hematoxylin and eosin (H&E) staining. Scale bar = 200 µm.

Given the potent inhibitory effects of Cyclo-C against PEDV *in vitro*, we next evaluated its therapeutic efficacy in a piglet infection model. To determine whether Cyclo-C functions as a post-entry antiviral agent, IPEC-J2 cells were treated with Cyclo-C at different time intervals following PEDV infection, and samples were collected at 24 hpi for immunoblot analysis. Continuous treatment (0–24 hpi) and post-infection treatment (2–24 hpi) significantly suppressed PEDV replication compared to corresponding control groups, whereas early-phase treatment (0–2 hpi) exhibited only marginal inhibitory effects (Fig. 8A). These findings indicate that Cyclo-C primarily acts as a therapeutic agent during established infection rather than at the viral entry stage.

To assess *in vivo* antiviral efficacy, PEDV-challenged piglets were administered different doses of Cyclo-C, and multiple clinical and virological parameters were evaluated. A schematic diagram of the experimental design is shown in Fig. 8B. All PEDV-infected piglets developed vomiting and diarrhea within 24 h post-challenge. In the PEDV-infected group, severe watery diarrhea and persistent vomiting were observed, with mortality occurring on days 4 and 5 post-infection. Piglets treated with a low dose of Cyclo-C exhibited milder symptoms, including loose stools; vomiting resolved after treatment, and diarrhea improved to semi-fluid or paste-like feces, although one piglet succumbed on day 5. In contrast, piglets treated with a high dose of Cyclo-C showed rapid symptom resolution, with feces gradually returning to normal consistency and no mortality observed throughout the study period. Mock-infected piglets displayed no clinical symptoms (Fig. 8C through E).

Post-mortem examination revealed striking differences in intestinal pathology. Mock-infected piglets exhibited normal intestinal morphology. In contrast, PEDV-infected piglets showed severe intestinal distension and marked thinning of the intestinal wall. Low-dose Cyclo-C treatment resulted in mild intestinal distension with accumulation of yellowish-green digestive fluid, whereas high-dose Cyclo-C treatment preserved normal intestinal architecture comparable to that of the mock group (Fig. 8E).

Rectal swabs were collected for viral RNA quantification by RT-qPCR. At 1 dpi, fecal viral loads reached approximately 10⁷ copies in all infected piglets. In the PEDV-infected group, viral loads increased gradually from 1 to 4 dpi. In the low-dose Cyclo-C group, viral loads decreased following treatment and remained consistently lower than those in the PEDV-infected group. In the high-dose Cyclo-C group, fecal viral RNA levels decreased after treatment and remained relatively stable throughout the observation period (Fig. 8F).

Viral loads in small intestinal tissues (duodenum, jejunum, and ileum) were also assessed. Compared with the PEDV-infected group, viral loads were significantly reduced in the low-dose Cyclo-C group and were profoundly suppressed in the high-dose treatment group (Fig. 8G). Consistently, Western blotting analysis of intestinal tissues showed a dose-dependent reduction in PEDV N protein expression following Cyclo-C treatment (Fig. 8H).

Histopathological examination further corroborated these findings. The PEDV-infected group exhibited severe villous atrophy, fragmentation, and dissolution of villous tips. The low-dose Cyclo-C group showed milder histopathological damage, although partial villous disruption persisted. Notably, the high-dose Cyclo-C group displayed intact villous architecture and normal intestinal morphology, comparable to that of mock-infected controls (Fig. 8I). Collectively, these *in vivo* results demonstrate that Cyclo-C confers dose-dependent protection against PEDV infection, reducing viral loads, alleviating clinical symptoms, preventing mortality, and preserving intestinal integrity.

## DISCUSSION

PEDV has caused recurrent outbreaks worldwide, resulting in substantial economic losses to the swine industry. Moreover, its potential for cross-species transmission raises concerns regarding possible public health implications (50, 51). Despite extensive efforts, currently available commercial vaccines provide limited protection against PEDV, particularly against emerging variants (52–54). Therefore, the identification of effective antiviral compounds represents a critical complementary strategy for controlling PEDV infection. Given that PEDV exploits host intracellular proteins to facilitate its replication and immune evasion, targeting host factors and elucidating their underlying mechanisms is of considerable practical and scientific importance. In this study, we identified Cyclo-C as a novel PEX13 agonist that effectively suppresses PEDV infection by modulating the peroxisomal autophagy pathway. Mechanistically, Cyclo-C upregulates PEX13 expression, thereby inhibiting PEDV-induced pexophagy through activation of the PI3K/AKT/mTOR signaling pathway. This suppression of pexophagy preserves peroxisomal integrity, stabilizes peroxisome-associated MAVS, enhances IFN-III production, and ultimately establishes a robust antiviral state.

Previous studies have demonstrated that PEDV NSP8 targets PEX13, promoting its degradation and inducing pexophagy, which allows the virus to evade peroxisome-mediated IFN-III responses (26). These findings raise an important question: can PEX13 serve as a viable antiviral target? Our results provide compelling evidence in support of this hypothesis. We demonstrate that PEX13 functions as a broad-spectrum restriction factor against multiple swine RNA viruses, including PEDV, PDCoV, and TGEV. The inhibition of both alphacoronaviruses (PEDV and TGEV) and a delta coronavirus (PDCoV) suggests that exploitation of peroxisomal function may represent a shared vulnerability among coronaviruses. These findings firmly establish the therapeutic relevance of PEX13 as a host-directed antiviral target for economically devastating swine diseases.

Building on this foundation, we conducted structure-based virtual screening to identify small-molecule agonists targeting PEX13. Among the five candidate compounds identified, only Cyclo-C lost its antiviral activity in PEX13 knockout cells, whereas the remaining compounds retained inhibitory effects on PEDV. This observation indicates that Cyclo-C exerts its antiviral function specifically through PEX13, while the other compounds likely act through alternative pathways. Molecular docking and molecular dynamics simulations further confirmed stable binding between Cyclo-C and PEX13. Consistently, Cyclo-C treatment resulted in a time-dependent increase in PEX13 protein levels, providing strong experimental support for this interaction.

Given our previous findings that PEDV NSP8 induces PEX13 degradation, we next investigated whether Cyclo-C could modulate the interaction between NSP8 and PEX13. Confocal microscopy revealed that Cyclo-C stabilized the subcellular localization of PEX13 during PEDV infection, thereby preventing NSP8 from recruiting PEX13 to the RTC sites on the ER. As a result, PEX13 escaped NSP8-mediated degradation, leading to sustained protein stability. These findings demonstrate that Cyclo-C counteracts PEDV-induced pexophagy by protecting PEX13 from viral manipulation.

Autophagy, including selective pexophagy, is tightly regulated by the PI3K/AKT/mTOR signaling pathway, with mTOR acting as a central negative regulator. PEDV infection suppressed phosphorylation of PI3K, AKT, and mTOR, thereby promoting pexophagy. Cyclo-C treatment effectively restored the phosphorylation levels of these kinases, whereas pharmacological inhibition of PI3K, AKT, or mTOR reversed both the anti-pexophagy and antiviral effects of Cyclo-C. These results indicate that Cyclo-C suppresses PEDV-induced pexophagy through activation of the PI3K/AKT/mTOR pathway downstream of PEX13 stabilization.

Pexophagy is the primary pathway for peroxisome turnover, and peroxisomes are essential platforms for IFN-III induction in intestinal epithelial cells. Inhibition of pexophagy by Cyclo-C preserved peroxisomal integrity, as evidenced by restored levels of peroxisomal markers such as PMP70 and catalase. This preservation stabilized peroxisome-associated MAVS, leading to enhanced IFN-III expression. Importantly, blockade of the PI3K/AKT/mTOR pathway abolished Cyclo-C–induced increases in MAVS and IFN-III, confirming that this signaling axis is essential for its antiviral mechanism. Collectively, these findings demonstrate that the ultimate antiviral effect of Cyclo-C is mediated through reinforcement of the peroxisome–MAVS–IFN-III signaling axis. Notably, the *in vivo* efficacy of Cyclo-C in PEDV-infected piglets strongly corroborates these mechanistic findings. Cyclo-C treatment alleviated clinical symptoms, reduced viral loads, and preserved intestinal architecture in a dose-dependent manner. The effectiveness of post-infection treatment and the absence of mortality in the high-dose group highlight its promising therapeutic potential for clinical application.

**Fig 9:**
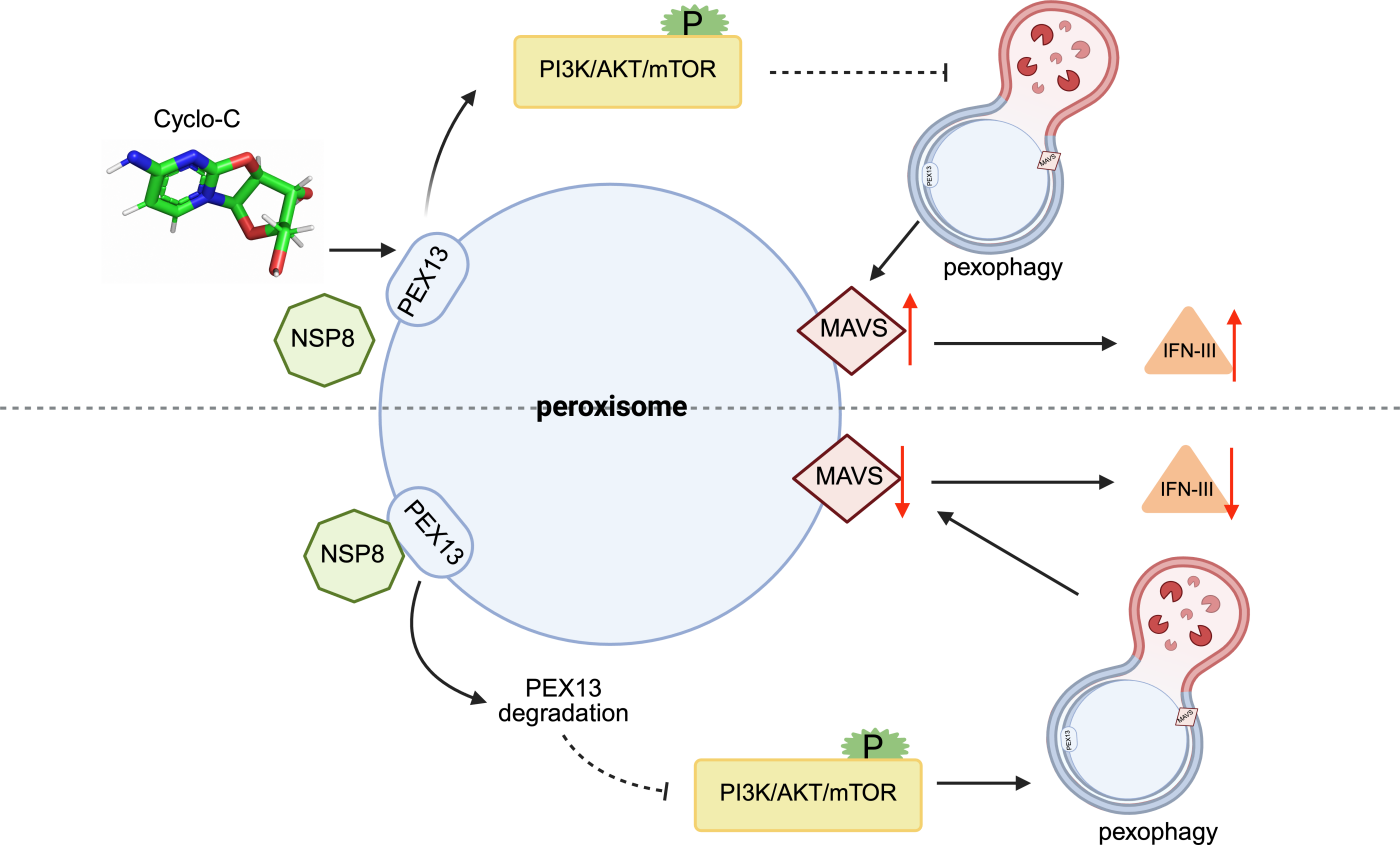
Schematic diagram illustrating the mechanism by which Cyclo-C stabilizes PEX13, inhibits peroxisome autophagy, and promotes IFN-III production. Cyclo-C promotes PEX13 expression, inhibiting PEDV NSP8-mediated PEX13 degradation, thereby activating the PI3K/AKT/mTOR pathway, suppressing pexophagy, and enhancing MAVS and IFN-III expression.

In conclusion, this study advances PEX13 from a viral target of immune evasion to a tractable host-directed antiviral defense factor. We identify Cyclo-C as a first-in-class PEX13 agonist that counteracts PEDV-mediated immune suppression by restoring peroxisomal integrity and innate immune signaling (Fig. 9). By reactivating the peroxisome–MAVS–IFN-III axis, Cyclo-C establishes a potent antiviral state that is inherently less susceptible to viral resistance than direct-acting antivirals. Future studies will focus on optimizing the pharmacokinetic properties of Cyclo-C and evaluating its efficacy against a broader spectrum of viruses that exploit peroxisomal immunity, thereby opening new avenues for host-directed antiviral therapy.

## MATERIALS AND METHODS

### Cell lines, viruses, and plasmids

Vero E6 cells (ATCC CCL-81) and HEK293T cells (ATCC CRL-3216) were cultured in Dulbecco’s modified Eagle’s medium (DMEM; Corning, USA) supplemented with 10% fetal bovine serum (FBS; Gibco, USA) and 50 IU/mL penicillin/streptomycin (30-002-CI, Corning, USA). IPEC-J2 is a continuous line of epithelial cells derived from the jejunum of a 12-h-old, colostrum-unsuckled, mixed breed piglet (55) and was cultured in DMEM (Corning, USA) supplemented with 10% FBS. All cell lines were maintained at 37°C in a humidified incubator containing 5% CO₂.

The PEDV variant strain MSCH (GenBank accession no. MT683617) was isolated, maintained in our laboratory, and propagated in Vero E6 cells and IPEC-J2 cells in the presence of 6 μg/mL trypsin (Sigma-Aldrich, USA), as previously described (56).

Plasmids encoding HA-tagged PEX13, RFP-GFP-SKL, and GFP-PEX13 were constructed in our laboratory using standard molecular cloning techniques. Expression plasmids encoding PEDV structural and nonstructural proteins were generated as described previously (57).

### Antibodies and chemicals

Rabbit antibodies against LC3, p62, MAVS, catalase, PMP70, and PEX13 were purchased from Proteintech (USA). Mouse anti-Flag and rabbit anti-phospho-PI3K (Tyr467/199) antibodies were obtained from Abmart (China). Mouse anti-PI3K was purchased from Selleck (USA). Rabbit antibodies against HA, mTOR, phospho-mTOR (Ser2448), AKT, and phospho-AKT (Ser473) were purchased from Cell Signaling Technology (CST, USA). Monoclonal antibodies against PEDV N protein and polyclonal antibodies against NSP8 were generated and preserved in our laboratory. Horseradish peroxidase (HRP)-labeled rabbit or mouse secondary antibodies were acquired from Bioss (China).

Cells were treated with dimethyl sulfoxide (DMSO), proteasome inhibitors, or autolysosome inhibitors, ensuring that the final concentration of DMSO did not exceed 0.1%. PI-103, LY294002, and Torin-1 were purchased from Selleck (USA). Cyclo-C used for *in vitro* experiments was purchased from TargetMol (Shanghai, China), whereas Cyclo-C used for animal experiments was obtained from Macklin (China).

### Western blotting analysis

Cells were lysed on ice for 30 min using radioimmunoprecipitation assay (RIPA) lysis buffer (Beyotime, China). The lysates were then separated by SDS-PAGE and transferred onto nitrocellulose (NC) membranes. Membranes were blocked with 10% nonfat milk in PBST for 2 h at room temperature, followed by incubation with the appropriate primary antibodies overnight at 4°C. After washing with PBST, membranes were incubated with HRP-conjugated secondary antibodies for 1 h at room temperature. Protein bands were visualized using an enhanced chemiluminescence (ECL) kit (Tanon, China), and specific bands were analyzed using a Tanon 5200 chemiluminescence imaging system (Tanon, China).

### RT-qPCR

Cells were washed once with PBS, and RNA was extracted using a Total RNA Kit I (Omega Bio-Tek, USA) according to the manufacturer’s instructions. For reverse transcription, 1 μg of total RNA was reverse transcribed using HiScript qRT SuperMix (Vazyme, China). Reactions were conducted using an ABI QuantStudio 6 System (Applied Biosystems, USA) with AceQ qPCR SYBR Green Master Mix (Vazyme, China) following the provided instructions.

Relative expression levels of the target genes were calculated using the comparative cycle threshold (CT) method. Host and PEDV expression levels were normalized to the housekeeping gene β-actin. Primer sequences used for RT-qPCR are listed in Table 1.

**TABLE 1:** Real-time PCR primer sets for cytokine genes used in this study

Primer	Forward primer sequence (5′–3′)	Reverse primer sequence (5′–3′)
PEDV N	CGTACAGGTAAGTCAATTAC	GATGAAGCATTGACTGAA
PPDCoV	CTTAACTCCCCCATCAAACCC	TAACCCAAGTAACACGAGTGG
TGEV	CAATTCCCGTGGTCGGAAGA	TTTACGTTGGCCCTTCACCA
β-Actin	CTCCATCATGAAGTGCGACGT	GTGATCTCCTTCTGCATCCTGTC
IFN-λ1	GGTGCTGGCGACTGTGATG	GATTGGAACTGGCCCATGTG
IFN-λ3	ACTTGGCCCAGTTCAAGTCT	CATCCTTGGCCCTCTTGA
IFN-λ4	GCTATGGGACTGTGGGTCTT	AGGGAGCGGTAGTGAGAGAG

### Virus titration

Vero cells were cultured in 96-well plates and allowed to reach confluence overnight. The cells were then incubated with 100 μL of serially diluted supernatant containing PEDV for 2 h at 37°C and 5% CO2. Count the number of wells with lesions under the microscope 36 h after infection. Data are presented as TCID_50_ from quadruplicate samples.

### Cytotoxicity assay

The test compounds were added to the corresponding cells and incubated for 24 h at 37°C. Cell viability was tested using an enhanced Cell Counting Kit-8 (CCK-8; Vazyme, China) following the manufacturer’s instructions. An equal volume of DMSO was used as the control. The CC_50_ was calculated using GraphPad Prism 7.0 software.

### Antiviral activity assay

To investigate the antiviral effects of Cyclo-C on PEDV (MOI = 1), IPEC-J2 cells were infected with the virus. After incubation at 37°C for 1 h, the supernatants were removed, and DMEM supplemented with 6 μg/mL trypsin containing different concentrations of Cyclo-C (4, 10, 50, 100, 150, and 200 μM) or an equal volume of DMSO was added. Finally, cells and supernatants were collected at the indicated time points, and the virus titer, number of virus-infected cells, viral protein levels, and viral RNA levels were evaluated by Western blotting analysis and RT-qPCR, respectively. The fluorescence intensity was determined by ImageJ software, and the 50% effective concentration (IC_50_) was estimated by GraphPad Prism 7.0 software.

### Virtual screening

The structure of PEX13 was simulated by AlphaFold 3 and then used for virtual screening. Marketed drugs (~1,700 compounds) from the FDA were screened using the molecular docking program Vina. A semi-flexible docking protocol was used to perform the calculations. The global search exhaustiveness value was set to 50. The maximum energy difference between the optimal binding mode and the worst case was set to 5 kcal/mol.

### Molecular dynamics simulations

It was parameterized using the GROMOS87/GROMOS96 force field, with topology files generated by PRODRG 2.5. Simulations were conducted with the Gromacs package using the GROMOS96 43A1 force field. The simple point charge 216 (SPC 216) model of water was used to solvate the protein in a periodic dodecahedron box extending 10 Å from the nearest protein atom. The solvated system was then neutralized with Na^+^ and Cl^−^ ions, minimized by the steepest descent method (50,000 steps), and equilibrated with a 100-ps constant volume (NVT) simulation. The production runs were conducted in a constant pressure ensemble (NPT). The temperature was set to 300 K and controlled with V-rescale. Long-range electrostatic interactions were treated with the particle-mesh Ewald (PME) method. The pressure was coupled to 1 bar using the Parrinello-Rahman method. All bond lengths were constrained with the LINear Constraint Solver (LINCS) algorithm. A cut-off of 14 Å was used to calculate short-range van der Waals and electrostatic interactions. The total simulation time was 200 ns. RMSD, the number of hydrogen bonds, and Gibbs Free Energy Landscape between Cyclo-C and active pockets of PEX13 were analyzed to judge binding stability and convergence.

### Lentivirus production and transduction

Lentiviruses were generated by co-transfection of HEK293T cells with the packaging plasmids pMD2.G (Addgene #12259), psPAX2 (Addgene #12260), and plentiCRISPRv2-sgRNA (sgPEX13 5′-CACCGGGGAGACCCGCCGGATTCCG-3′, 5′-AAACCGGAATCCGGCGGGTCTCCCC-3′) using polyethylenimine (PEI; Yeasen, China). The culture medium was replaced at 12 h post-transfection. Viral supernatants were collected at 48 and 60 h post-transfection and stored at −80°C. Target cells were transduced with lentiviral particles in the presence of 10 μg/mL polybrene. PEX13-KO cells were generated from single-cell clones, which were selected by limiting dilution following puromycin selection, and knockout was confirmed by Western blotting.

### Co-immunoprecipitation assay

Cells were transfected with plasmids for 24 h and lysed with NP-40 cell lysis buffer supplemented with PMSF (protease inhibitor, Beyotime). Cell lysates were centrifuged and incubated with anti-Flag magnetic beads (Sigma, USA) and anti-HA magnetic beads (CST, USA). After washing with PBST, immunocomplexes were eluted using 50 mM glycine buffer (pH 2.8) and subjected to Western blotting with the indicated antibodies.

### Confocal microscopy

Cells were seeded onto 15-mm glass-bottom dishes (Nest Biotechnology, China). Cells were fixed with 4% paraformaldehyde for 10 min at room temperature, permeabilized with 0.1% Triton X-100 for 10 min, and blocked with 2% bovine serum albumin (BSA) in PBS for 1 h. Cells were incubated with primary antibodies overnight at 4°C, followed by incubation with Alexa Fluor 488–conjugated secondary antibodies (Proteintech, China) for 1 h at room temperature. Nuclei were counterstained with DAPI (Biosharp, China) for 10 min. After washing with PBST, images were acquired using a Nikon A1 confocal microscope (Japan). Two or three fluorescence channels were recorded sequentially or simultaneously to avoid signal overlap.

### Animal experiment

Three-day-old colostrum-deprived piglets (negative for TGEV, PEDV, and PDCoV) were randomly divided into four groups: the PBS group, the PEDV-infected group, the PEDV-infected group with low-dose Cyclo-C (1.35 mg/kg), and the PEDV-infected group with high-dose Cyclo-C (4 mg/kg). Each group consisted of three piglets. The challenge groups were orally inoculated with 2 mL of DMEM containing 2 × 10⁶ PFU PEDV, while the control group was orally inoculated with an equal volume of DMEM medium. At 24 h post-challenge, the corresponding drugs were administered via intramuscular injection twice a day for four consecutive days. The fecal status of the piglets was scored daily according to the following criteria: 0 points (solid), 1 point (paste-like), 2 points (semifluid), and 3 points (liquid). Rectal swab samples were collected from the piglets every day after the challenge, and then RT-qPCR was performed to detect the daily PEDV shedding amount. On the fifth day after virus infection, all the surviving piglets were humanely euthanized, followed by necropsy. The intestinal segments of piglets from each group were collected for HE staining, RT-qPCR to detect the intestinal virus load, and Western blotting to detect the content changes of PEDV-N.

### Pathological examination of intestinal sections

At necropsy, the duodenum, jejunum, and ileum tissues were collected and fixed in formalin for histological examination. The dehydrated tissues were cleared with xylene, embedded in paraffin wax, sectioned, and mounted onto slides. These slides were then subjected to H&E staining.

### Statistical analysis

All experiments were performed independently at least three times. Data were analyzed using one-way or two-way analysis of variance (ANOVA) followed by Tukey’s post hoc test for multiple comparisons using GraphPad Prism software (GraphPad Software Inc., San Diego, CA, USA). Results are presented as mean ± standard deviation (SD). A *P* value of <0.05 was considered statistically significant (*), while *P* values of <0.01 (**), <0.001 (***), and <0.0001 (****) were also considered significant, and the notation “ns” indicates non-significant differences.

## Data Availability

All relevant data generated in this study are present within the article.
